# Exposing intracellular molecular changes during the differentiation of human-induced pluripotent stem cells into erythropoietin-producing cells using Raman spectroscopy and imaging

**DOI:** 10.1038/s41598-022-24725-1

**Published:** 2022-11-28

**Authors:** Mika Ishigaki, Hirofumi Hitomi, Yukihiro Ozaki, Akira Nishiyama

**Affiliations:** 1 Institute of Agricultural and Life Sciences, Academic Assembly, Shimane University, 1060 Nishikawatsu, Matsue, Shimane 690-8504 Japan; 2 Raman Project Center for Medical and Biological Applications, Shimane University, 1060 Nishikawatsu, Matsue, Shimane 690-8504 Japan; 3 Department of iPS Stem Cell Regenerative Medicine, Kansai Medical University, 2-5-1 Shin-Machi, Hirakata, Osaka 573-1010 Japan; 4 School of Biological and Environmental Sciences, Kwansei Gakuin University, 1 Gakuen-Uegahara, Sanda, Hyogo 669-1330 Japan; 5 Department of Pharmacology, Faculty of Medicine, Kagawa University, 1750-1 Miki-Cho, Kita, Kagawa 761-0793 Japan

**Keywords:** Stem cells, Imaging studies

## Abstract

The objective of this study was to explore intracellular molecular changes during the differentiation of human-induced pluripotent stem cells (iPSCs) into erythropoietin (EPO)-producing cells using Raman spectroscopy and imaging. Raman imaging data of fixed cells at four stages of cell differentiation were analyzed by a partial least squares (PLS) regression model, and the variations in the intracellular molecular compositions with cell differentiation were investigated. As a result, three biomarkers characterizing the cell phases were identified: dimethyl sulfoxide (DMSO), fatty acids with a low grade of unsaturation, and glycoproteins. The uptake of DMSO by EPO-producing cells, which was added into a culture medium as an inducer for cell differentiation, was detected, and the increase in unsaturated fatty acid concentrations was revealed that lipid metabolism changed over the course of cell differentiation. The decrease in the glycoprotein concentration after the cell phase during which iPSCs differentiated into EPO-producing cells was also made clear. Raman imaging successfully visualized chemical images of these three biomarkers in two dimensions, where the biomarker concentrations independently varied during cell differentiation. These results demonstrated the application potential of the proposed method to regenerative medicine for monitoring cell differentiation and discriminating cell maturation in situ at the molecular level.

## Introduction

Pluripotent stem cells have the ability to differentiate into any type of cell and proliferate infinitely^1,2^. Humans have developed techniques to artificially produce pluripotent stem cells by reprogramming somatic cells called induced pluripotent stem cells (iPSCs)^3^. These iPSCs can be used for cell therapy, drug screening, toxicology, and disease modeling^4–6^ and have therefore attracted keen interest for developing novel pharmaceuticals and application to regenerative medicine.

Erythropoietin (EPO) is a glycoprotein that regulates the production of red blood cells and is produced in the adult kidney^7^. Recombinant human EPO (rhEPO) is very effective in the treatment of renal anemia but is associated with problems such as the need for frequent administration and refractory cases. EPO is generated by culturing human EPO-producing cells and could be used to solve these problems. Although Obara et al. identified EPO-producing cells in mouse kidneys^8^, a successful method for culturing EPO cells has not been developed to date. Thus, monitoring EPO produced by the differentiation of iPSCs into EPO-producing cells could provide a means of developing a novel physiological therapy for renal anemia. We succeeded in generating cells expressing EPO from mouse and human iPSCs and ESCs by modifying previously reported protocols for differentiation of cells into hepatic lineages, ending a long period during which no method was available to differentiate EPO-producing cells directly from these stem cells^9^.

For the practical application of these stem cells in the near future to medical care and the development of pharmaceuticals, it is essential to establish a noninvasive real-time cell evaluation method that can be used in situ. Existing methods for cell evaluation, gene expression, protein composition, and lipid components require analysis by an RNA assay, fluorescent staining, electrophoresis, and gas chromatography, all of which are destructive. As a safe and stable cell supply is becoming increasingly important for the usage of stem cells, the development of a nondestructive method of evaluating cells without staining during the course of cell differentiation from stem cells into any type of somatic cell is urgently needed.

Raman spectroscopy is a type of vibrational spectroscopy that can be used to obtain information in situ in a nondestructive and label-free manner for substances such as proteins, lipids, and nucleic acids, including the molecular compositions, relative concentrations, and the molecular structure. Raman imaging can be used to obtain two- and/or three-dimensional chemical images of uneven biomolecular distributions and concentration variations^10^. Raman spectroscopy and imaging are very useful tools for in situ bioanalysis and have been used by many research groups for various biological and medical applications^11–19^. An example of a similar application of Raman spectroscopy to stem cell analysis as to the present study is the study of Notingher et al., which was the first report of the differentiation of mouse embryonic stem cells (ESCs) using this method^16^. The RNA concentration in ESCs and spontaneously differentiated cells was found to be a significant biomarker for discriminating differentiation of ESCs. Germond et al. used Raman spectroscopy to monitor the reprogramming process of neuronal cells that were differentiated from mouse ESCs^17^. The different states of cells during the course of reprogramming were identified based on the differences of Raman spectra, and ESCs and reprogrammed cells were distinguished each other. Chan et al. targeted the differentiation of human ES cells (hESCs) into cardiomyocytes (CMs)^18^. hES cells have higher contents of DNA and RNA than hESC-derived CMs and human fetal left ventricular CMs, and a principal component analysis (PCA)-linear discriminant analysis (LDA) algorithm can be used to discriminate these three types of cells with high accuracy. Tan et al. compared the Raman spectra of human-induced pluripotent stem cells (hiPSCs), hESCs and nonspecifically differentiated progeny of hESCs^19^. The spectral patterns of differentiated hESCs were significantly different from those of other cells, and slight differences between hiPSCs and hESCs were detected based on the glycogen-like spectral component.

The objective of the present study was to explore intracellular molecular changes during the cell differentiation of hiPSCs into EPO-producing cells using Raman spectroscopy and imaging. The observed changes in Raman spectra obtained from fixed cells over the course of cell differentiation were analyzed. Complex Raman spectra of cells with many overlapping signals from proteins, lipids, saccharides, and other substances were disentangled into independent molecular components that varied in concentration with the cell phases, and the corresponding concentration profiles were obtained by making full use of a partial least squares (PLS) regression analysis. The band intensity of the C-S-C stretching modes was found to increase as the iPSCs differentiated into EPO-producing cells, which is a sign of epigenetic variation due to the addition of DMSO to the culture medium. In addition, the intracellular metabolism of lipids was clearly found to change with cell differentiation because an increase in the concentration of fatty acids with a low degree of unsaturation was detected. Furthermore, the glycoprotein concentration decreased during the phase just after the differentiation into EPO-producing cells. This result suggests the maturation of EPO-producing cells could be identified by detecting a decrease in the concentration of glycoprotein in the differentiated cells.

The considerable success in capturing intracellular metabolic and epigenetic changes over the course of cell differentiation provides new insights into the cytology of stem cells. Furthermore, the key molecules for the cell phases identified in the present study can serve as useful biomarkers for developing a steady and safe monitoring method for cell differentiation in regenerative medicine. The proposed method is expected to be applicable for monitoring EPO produced by differentiated iPSCs for use in the development of pharmaceuticals.

## Materials and methods

### Cell culture

Human iPS cells (253G1) were purchased from Riken (Wako, Japan). The cells were cultured using an established protocol in a feeder-free manner. The detailed procedure for the public protocol was provided by the Center for iPS Cell Research and Application, Kyoto University^20^. After confirming that the culture of iPSCs was stable, the iPSCs were differentiated into EPO-producing cells in a 24-well plate with 5 $\times$ 10^4^ cells per well. Table S1 shows the substances required for differentiation of hiPSCs into EPO-producing cells, which varied with the cell stages ranging from iPSC cells to Stage 2 (Fig. S1a). The cells were incubated under 5% CO_2_ at 37 °$\mathrm{C}$.

In the present study, the cells were fixed for four phases: Phases I, II, III, and IV (Fig. S1a). Since iPSCs are sometimes sensitive to external stimulations, photo stimulation by laser irradiation may cause differentiation into cells with slightly different properties from EPO-producing cells. To eliminate such possibilities and to explore the molecular changes with cell differentiation into EPO-producing cells as a “standard”, the fixed cells were studied. The cells were iPSCs during Phase I and had differentiated into EPO-producing cells by the final stage, Phase IV. In addition to the phases in which the cells were iPSCs (Phase I) and EPO-producing cells (Phase IV), the cells were also fixed at two intermediate points between Phase I and IV, that is, Phase II and III. During Phase II, the cells had almost differentiated into endoderm cells. Images of the cells during the four phases are shown in Fig. S1b–d.

Cell fixation was carried out by adding a 4% paraformaldehyde solution to the culture medium, which was allowed to stand for approximately 1 h, followed by washing twice with PBS, and drying. An immunostained image of the cells during Phase IV obtained using anti-hEPO, Alexa 594, and Hoechst proved that EPO was secreted because the cells were dyed red (Fig. S1e). The detailed information about cell culture is summarized in Supporting Information (SI) 1.

Experiments performed on hiPSCs were approved by the Ethics Committees of Kyoto University and Kagawa University. Informed consent was obtained from the donor from whom the hiPSCs were derived according to the guidelines of the Declaration of Helsinki. All methods were performed in accordance with institutional guidelines.

### Raman measurement and imaging

Raman measurements on the fixed cells were carried out using an inverted microscope Raman system, XploRA INV (HORIBA Ltd., Kyoto, Japan) operated by LagSpec6 software (HORIBA Ltd., Kyoto, Japan), which consisted of a 785-nm diode laser, a spectrometer, a 600-g/mm grating, a CCD camera, and a microscope (Eclipse Ti2 Inverted Microscope, Nikon Co., Tokyo, Japan) with a 60$\times$ water immersion objective lens (Plan Apochromat, NA = 1.20, Nikon). The measured Raman spectra were calibrated using the peak for silicon. Raman spectra were acquired in imaging mode using 40 mW at the sampling point by dripping water between the objective lens and a quartz dish. Imaging data with a 20 μm $\times$ 20 μm area were obtained using a 1-μm step in 10 s (5 s $\times$ 2 times). Raman images were constructed by plotting the characteristic Raman band intensity depending on cell differentiation and the score plots from the PLS regression analysis.

The autofluorescence background was removed using 5th-order polynomial fitting using a calculation program that was developed in-house. The spectral intensity was normalized by the intensity of the standard peak at 1001 cm^−1^ for phenylalanine to obtain a peak height of unity. The argument for selecting the normalization method for Raman spectra is provided in SI 2. Ten imaging data for each phase were acquired from different ten points of different two dishes, and the mean Raman spectra for each phase were calculated using 4000 preprocessed spectra.

### Multivariate analysis

Multivariate analysis was performed using PLS regression with the chemometrics software Unscrambler X 10.3 (Camo Analytics, Oslo, Norway). PLS regression analysis can be used to construct a linear regression model between measurement data ($X$*)* and a response variable ($Y$), such as the concentration and density of a material, respectively^21^.

If the measured data contain the information represented by the response variable, regression coefficients ($B$) exist that satisfy Eq. (1).

1 $$\begin{array}{c}Y=XB+E.\end{array}$$

Here, $E$ is a residual, and all variables are generally expressed as matrices. $X$ and $Y$ are factorized into optimal loadings and scores to satisfy Eq. (1). Thus, the loadings and scores express how the data are projected onto the model components. For a linear regression model with high calibration accuracy, the root mean square error (RMSE) between the model predictions $Y{^{\prime}}$ and the measured values *Y* is small, and the coefficient of determination (*R*^2^) is close to 1. In this study, the concentration profile *Y* was not known. Thus, PLS analysis was performed by using artificial concentration profiles to extract the components that were correlated with the concentration profile, such as Phase (I, II, III, IV) = (1, 0, 0, 0) and = (0, 1, 0, 0), and a calibration model was built using the leave-one-out cross (LOOC) validation method. The LOOC method consisted of excluding one spectrum for use in validation, and the calibration model was built using the remaining data. The excluded spectrum was applied to the calibration model, and the predicted residual was calculated. This process was repeated until all the spectra had been excluded once. The accuracy of the models was assessed in terms of the squared correlation coefficient (*R*^2^).

### Sample preparation

Raman spectra for glucose, glycogen, and EPO aqueous solutions were obtained for reference. Glucose (045-31162, FUJIFILM Wako Pure Chemical Co., Japan) and glycogen (074-05561, FUJIFILM Wako) were dissolved in ultrapure water (214-01301, FUJIFILM Wako) to prepare 200 mM stock solutions. A total of 500 units of recombinant human EPO (519-26401, FUJIFILM Wako) were diluted in 2 μL of 0.05 mol/L Tris–HCl buffer solution (208-14691, FUJIFILM Wako). The prepared solutions were enclosed in glass capillaries (68402845, BRAND GMBH + CO KG, Wertheim, Germany), and Raman measurements were carried out at a 532-nm excitation wavelength with an 1800-g/mm grating and a 40$\times$ objective lens (S Plan Fluor ELWD, NA = 0.6, Nikon Co.). To preprocess the measured spectra, the spectra of the glass capillary and solvent were subtracted as backgrounds.

### Statistical analysis

A comparative analysis of multiple groups was performed using one-way analyses of variance (ANOVA) with Tukey’s method. The Student’s *t*-test was adopted to evaluate whether there were statistically significant differences between the mean values of two groups. The threshold *p* value for judging the significant differences was set to 0.05.

## Results and discussion

### Mean Raman spectra

**Figure 1:**
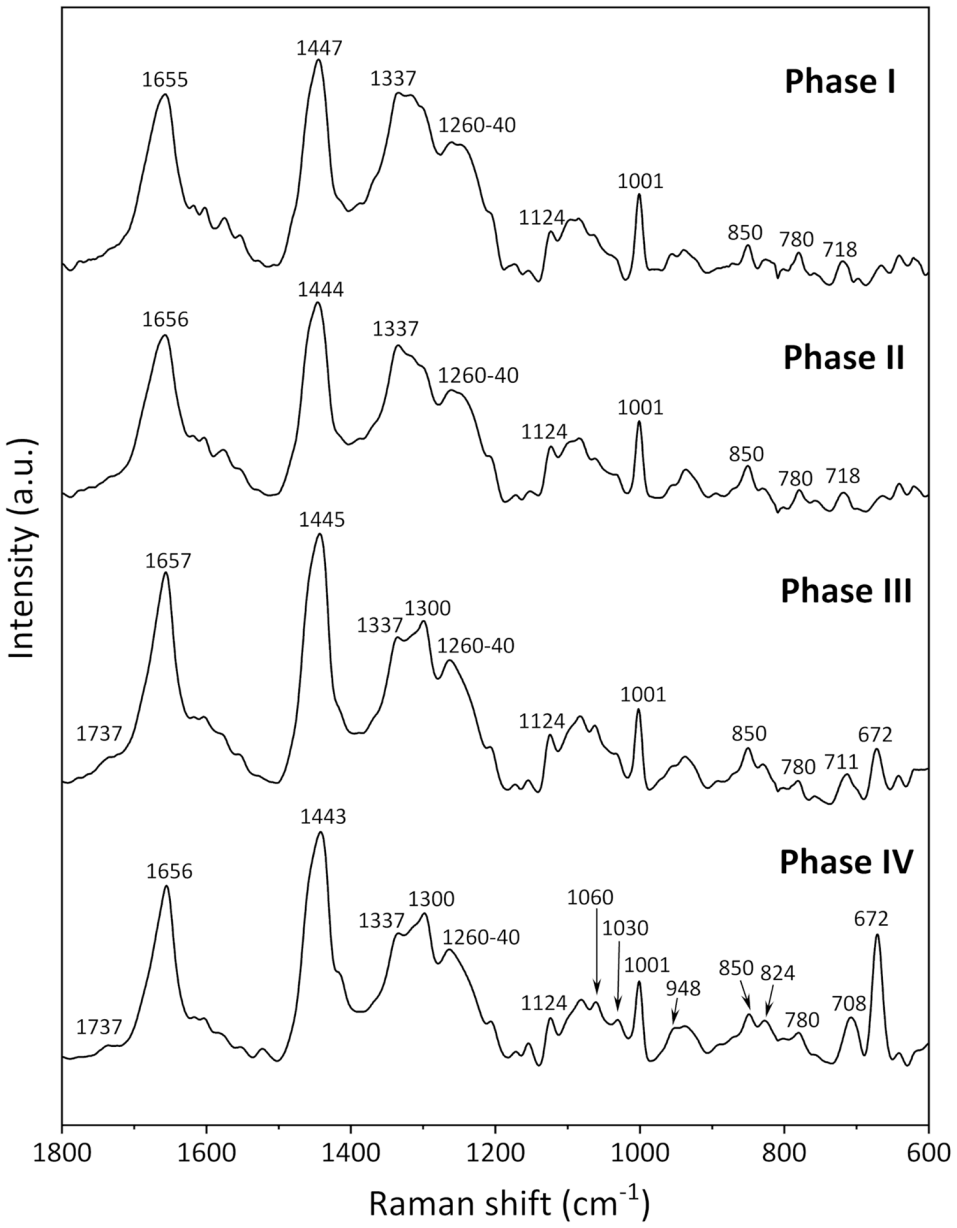
Mean Raman spectra in the 1800–600 cm^−1^ region for fixed iPSCs obtained for Phases I, II, III, and IV.

Figure 1 shows the mean Raman spectra in the 1800–600 cm^−1^ region obtained for Phases I, II, III, and IV. These spectra were calculated based on two-dimensional imaging data and therefore have a high signal-to-noise ratio. Many overlapping bands from various biomolecules, such as proteins, lipids, and DNA/RNA, are detected in this wavenumber region, the so-called “fingerprint region” of molecules. The peak at 1001 cm^−1^ is derived from the ring breathing mode of phenylalanine^22^, and its peak height was used as an internal standard to normalize the Raman spectra. In the spectra, the bands at 1655 cm^−1^ correspond to the amide I modes of proteins and the C=C stretching modes of lipids; the bands at 1447 cm^−1^ arise from the CH deformations of lipids, proteins, and carbohydrates; and those at 1337–1300 cm^−1^ were assigned to the CH_3_/CH_2_ twisting and bending modes of proteins and lipids^22^. The broad band in the 1260–1240 cm^−1^ region arises from the ring breathing modes of DNA/RNA (T, A), amide III of proteins, and bending mode of the =C–H group of lipids. The peak at 1124 cm^−1^ originates from C–N stretching of proteins, and the doublet at 850 and 824 cm^−1^ is attributed to the tyrosine residue, which results from Fermi resonance between a ring breathing mode and the overtone of the out-of-plane ring bending vibration of tyrosine^16,22,23^. The band at 780 cm^−1^ is attributed to the ring breathing modes of DNA/RNA (U, T, C), and the band at 718 cm^−1^ derives from the symmetric stretch vibration of the choline group CN^+^(CH_3_)_3_ of lipids^16,18,22^. The detailed assignment of the bands is summarized in Table S2.

There were systematic changes in the mean spectra obtained for each phase of cell differentiation from iPSCs to EPO-producing cells. For example, in the spectrum for Phase III, the band at 1655 cm^−1^ became sharp, and the center of the band positions at 1337–1300 and 1260–1240 cm^−1^ shifted to lower and higher frequencies, respectively, compared to the spectrum for Phase II. Furthermore, the band intensities at 1060, 1030, and 948 cm^−1^ in the spectra increased with cell differentiation. Changes in the ratio of the band intensities of the tyrosine doublet at 850 and 824 cm^−1^ were observed. Notably, prominent bands were observed at 708 and 672 cm^−1^ in the Phase III and IV spectra that were more intense in the Phase IV spectrum than the Phase III spectrum. These doublet peaks were assigned to asymmetric and symmetric C-S-C stretching vibrations, respectively^24,25^. These systematic spectral changes with cell differentiation can be interpreted to reflect the variations in the intracellular molecular composition characteristic to each cell phase. Thus, the variations in the cell metabolism and concentrations of key substances for inducing differentiation could be identified by an in-depth analysis of these spectral changes. Thus, we focus on the two bands at 708 and 672 cm^−1^ that are particularly interesting in relation to cell differentiation, and the corresponding cell functions and dynamics are discussed in the following subsections.

### Candidates for molecules with the C-S-C linkage

There are two candidates for molecules with the C-S-C linkage: DMSO and sulfur-containing amino acids. The first candidate is derived from DMSO added into a culture medium during Phase III and Phase IV (Table S1). The Raman spectrum of DMSO shown in Fig. S2 exhibits two characteristic bands at 702 and 672 cm^−1^ from antisymmetric and symmetric C-S-C stretching vibrations, respectively^24,25^ and is in good agreement with the spectral variations associated with cell differentiation. As the intensities of these two bands in the DMSO spectra are extremely high (Fig. S2), the DMSO concentration detected as an intracellular component during Phase III and Phase IV is likely to be very low even though the bands are strongly detected (Fig. 1). Generally, DMSO has been used both as a cryoprotectant and to induce differentiation of cultured cells^26–29^. It is well known that DMSO can promote or suppress the differentiation of cultured cells because of DNA methylation and histone modification, without changing the DNA base sequences^30–32^. For example, Iwatani et al. reported that DMSO affected the epigenetics of mouse embryonic stem cells and embryoid bodies by upregulating two subtypes of DNA methyltransferases and altering DNA methylation profiles^31^. Hay et al. reported the effect of DMSO treatment on inducing human embryonic stem cells into hepatic progenitor cells by histone acetylation^32^. Furthermore, the method used in the present study to induce differentiation of iPS cells to EPO-producing cells involves DMSO addition into the culture medium at the cell stage (Table S1) based on the results of a thorough investigation of the efficiency of inducing differentiation^20^. Deductive reasoning based on the aforementioned results leads us to expect that DMSO should induce the differentiation of iPSCs into EPO-producing cells by modifying epigenetics. That is, the changes in the two bands at 708 and 672 cm^−1^ with cell differentiation suggest that DMSO is taken up by cells and induces epigenetic changes.

The second possibility for the intense C-S-C Raman bands is to detect an increase in the concentrations of sulfur-containing amino acids with cell differentiation. Cysteine and methionine are general amino acids containing a sulfur atom. The molecular structures of cysteine and methionine contain C-S and C-S-C bonds, respectively, and the Raman spectra these amino acids exhibit a few peaks associated with their molecular structures in the 750–600 cm^−1^ region, as shown in Fig. S3^22,33^. However, the band positions of the pure amino acids (Fig. S3) were slightly different from those observed in the spectra obtained for Phase III and Phase IV (Fig. 1), and it was difficult to identify these two bands as corresponding to the sulfur-containing amino acids. In addition, the DMSO spectrum has characteristic bands at 1016 and 948 cm^−1^ associated with S = O stretching and CH_3_ stretching vibrational modes, respectively^22,24,25^, and these bands appeared to increase in intensity with cell differentiation at 1030 and 948 cm^−1^, respectively (Fig. 1). Thus, it can be concluded that the two bands at 708 and 672 cm^−1^ corresponding to C-S-C stretching modes derive from DMSO and can be used as marker bands for assessing the differentiation of iPSCs into EPO-producing cells.

### Biomolecules that increased in concentration with differentiation

**Figure 2:**
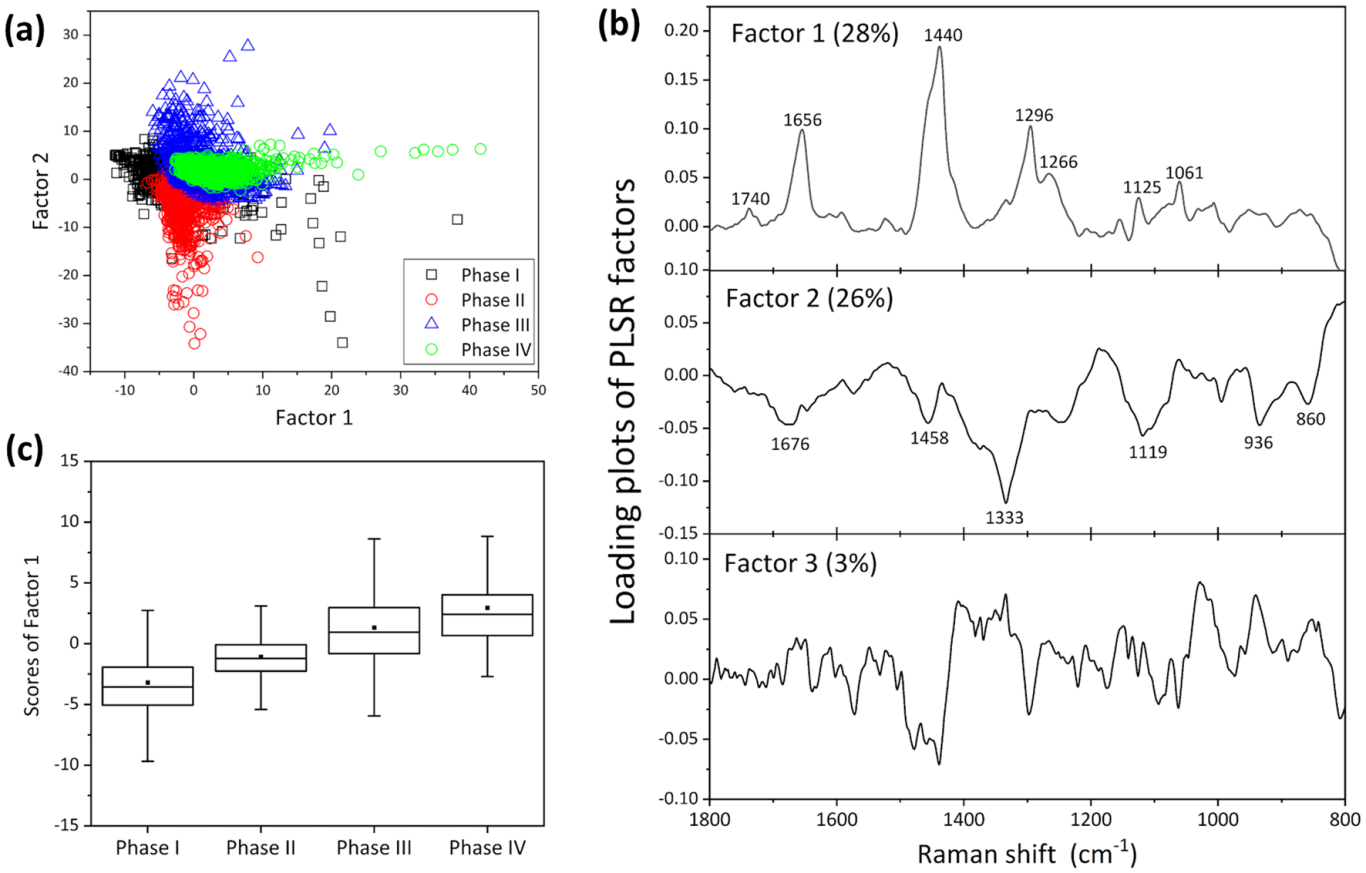
(**a**) Score plots of Factor 1 vs. Factor 2 and (**b**) loading plots of the main factors (up to Factor 3) for the PLS regression analysis. (**c**) Box plots of Factor 1. The box is expressed as the inter quarter range (IQR) = Q_3/4_ − Q_1/4_, and  is a mean value, where the central line is the mean value and the error bar shows the range between the minimum and maximum values.

In addition to the prominent changes in the Raman bands at 708 and 672 cm^−1^, several types of systematic spectral variations were observed over the course of cell differentiation. To identify the components that changed in concentration with cell differentiation, a PLS regression analysis was performed on the dataset for the 1800–800 cm^−1^ region, where the 800–600 cm^−1^ region containing the two strong bands due to the C-S-C stretching vibrational modes was eliminated. A PLS regression analysis can be used to construct a linear regression model between measurement data ($X$*)* and the concentration of the material of interest ($Y$), as has been detailed in the Materials and Methods section. First, the artificial concentration profiles were defined as corresponding to Phase (I, II, III, IV) = (0, 0, 0.5, 1) to determine the components that gradually increased in concentration with cell differentiation, and a calibration model was built using the leave-one-out cross validation method^34^. The maximum *R*^2^ for the calibration and validation models were 0.72 and 0.70, respectively, by including up to 3 factors. The score plots of Factor 1 vs. Factor 2 and the loading plots of the main factors (up to Factor 3) for the PLS regression are shown in Fig. 2a,b, respectively. The data points were grouped according to the cell phases (Fig. 2a). Figure 2c depicts the box plots of Factor 1. As the mean score values of Factor 1 increased with cell differentiation, Factor 1 was identified as the component reflecting the concentration profile. The significant differences at the 95% level in the mean scores of Factor 1 among the cell phases were determined by one-way ANOVA with Tukey’s method. The orders of all the *p* values obtained by the Student’s *t*-test for all combinations of two phases were significantly smaller than 0.01. The loading plot of Factor 1 exhibits the characteristic spectral pattern of fatty acids (Fig. S4, Table S3)^35–37^. In particular, based on the band at 1656 cm^−1^ corresponding to the C=C stretching vibration, Factor 1 yielded the spectral pattern characteristic of unsaturated fatty acids (Fig. 2b, Fig. S4). Beattie et al. analyzed the Raman spectra of an 18:n series of fatty acid methyl esters in the liquid state^35^. The following changes in the Raman spectrum were observed as the degree of unsaturation of the esters increased: the band intensity at 1657 cm^−1^ increased, the intensity ratio of the doublet band at 1305–1250 cm^−1^ changed, and the band intensity at 1280–1250 cm^−1^ corresponding to the =C–H bending mode increased beyond that of the band at 1305–1295 cm^−1^ derived from CH_3_/CH_2_ twisting and bending modes^35^. Based on these results, Factor 1 was found to exhibit a very similar spectral pattern to those of fatty acids with a low degree of unsaturation, especially oleic acid.

**Figure 3:**
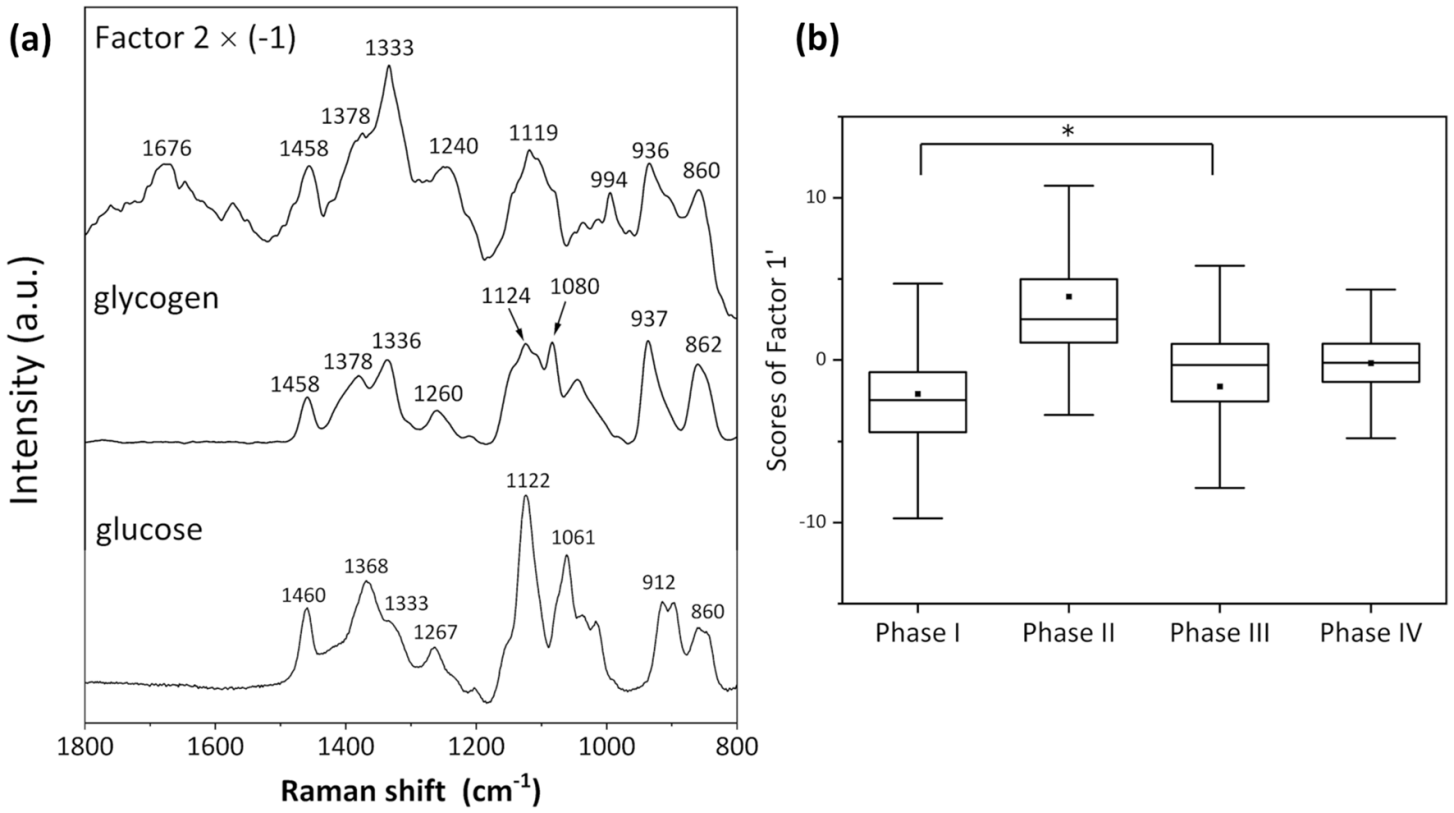
(**a**) Comparison of the inverse loading plots of Factor 2 (Factor 2 $\times$ (− 1)) against the Raman spectra of glucose and glycogen aqueous solutions. (**b**) Box plots of Factor 1ʹ showing the contribution from glycoproteins. The symbol * denotes a *p-*value smaller than 0.05, where the order of the *p* values for all other combinations between two phases were considerably smaller than 0.01.

The loading plot of Factor 2 exhibited some negative peaks. Excluding the broad peak at 1676 cm^−1^ originating from amide I, the complete spectral pattern of the loading plot of Factor 2 could be ascribed to that of saccharides. Thus, saccharides, especially glucose and glycogen in animal cells, are a candidate for the spectral component exhibiting the aforementioned band pattern. Figure 3a is a comparison of the inverse loading plots of Factor 2 (Factor 2 $\times \left(-1\right)$) against the Raman spectra of glucose and glycogen aqueous solutions. The band assignment of the saccharides is shown in Table S4^38–40^. Glycogen is a polysaccharide in which a large number of D-glucose molecules are polymerized by 1–4 (1–6) glycosidic bonds. The overall spectral pattern in the 1500–800 cm^−1^ region for [Factor 2 $\times$ (− 1)] was similar to that of glycogen (Fig. 3a). However, the band at 1676 cm^−1^ in the loading plot of Factor 2 did not appear in the spectra of glucose and glycogen. Furthermore, the band intensity at 1333 cm^−1^ appeared to be significantly higher in Factor 2 than that for glucose and glycogen. As the bands at 1676 and 1333 cm^−1^ correspond to amide I and amide III, respectively^22^, Factor 2 contains spectral information on both saccharides and proteins, namely, glycoproteins.

### Biomolecules that increased in concentration during the early phases of cell differentiation

To directly identify the components that increased in concentration during the early stages of cell differentiation (Phase I and Phase II), a PLS regression analysis was carried out using input profiles of assumed concentrations for Phase (I, II, III, IV) = (1, 0, 0, 0) and = (0, 1, 0, 0). As the value of *R*^2^ determined by assuming that a high concentration during Phase I subsequently decreased was low, no components were identified that increased in concentration during Phase I. By contrast, *R*^2^ was approximately 0.6 for any component with a high concentration during Phase II. The factor with the highest contribution to the PLS regression model (Factor 1ʹ) exhibited exactly the same spectral pattern as that of Factor 2 aforementioned, which was identified as the second component to build a model based on any concentrations that gradually increased during Phase III and Phase IV. Box plots for the Factor 1ʹ scores related to the glycoprotein components are shown in Fig. 3b. The results of the one-way ANOVA with Tukey’s method showed significant differences between all combinations of cell phases, and the order of the *p* values of the Student’s *t*-test between all combinations of two phases, except for those between Phase I and Phase III, were considerable smaller than 0.01. Namely, the concentration of glycoproteins was confirmed to be higher during Phase II than during the other phases.

EPO is a glycoprotein that regulates the production of red blood cells^7^. We previously reported that glycosylation of EPO modulated the activation and half-life of EPO degradation, and the difference in the aforementioned glycosylation patterns from those of two clinically available recombinant human EPOs appeared to affect the degree of effectiveness of EPO proteins^9^. Figure S5 shows the 1800–600 cm^−1^ region of the Raman spectra of EPO powder and EPO aqueous solution. The spectral patterns are very similar, although there are differences in the intensity ratios and broadening of some bands. Bands at 1088, 986, and 858 cm^−1^ corresponding to the C–C, C–O, and C–O–C stretching vibrational modes of saccharides were clearly observed in the spectrum of the EPO aqueous solution. However, the two bands at 986 and 858 cm^−1^ were shifted from those in the spectra of glucose and glycogen. This result was probably obtained because of the complex saccharide profiles of the sugar chain and the different glycosylation patterns between rhEPO and EPO produced by cultured cells. The band at 1660 cm^−1^ was assigned to amide I, whereas a band corresponding to amide III at approximately 1330 cm^−1^ was small. That is, the Raman spectrum of EPO exhibited the characteristics of both proteins and sugars even though the band of amide III was not clearly detected. The clear band at 1333 cm^−1^ for Factor 2 probably derived from the saccharides used as raw materials for glycosylation that were not linked with proteins. Thus, the concentrations of glycoproteins and saccharides, which were raw materials for EPO, appeared to decrease during Phase III and Phase IV because of EPO secretion.

### Raman imaging of fixed cells during four different phases of differentiation

The variations in the molecular compositions over the course of cell differentiation from iPS cells into EPO-producing cells were visualized in two dimensions by Raman imaging. Raman images were constructed by plotting the characteristic Raman band intensity that depended on cell differentiation and score plots for the PLS regression analysis. These plots can clearly show the spatial distribution of molecular compositions in a fluorescence-free manner without the use of staining.

In the previous sections, three molecular species were successfully identified as biomarkers for discriminating cell differentiation: DMSO, unsaturated fatty acids, and glycoproteins. Changes in the concentration and distribution of these components were visualized using the Raman band intensity for the C-S-C stretching vibration of DMSO and the concentration profiles of unsaturated fatty acids and glycoproteins determined by PLS regression analysis.

**Figure 4:**
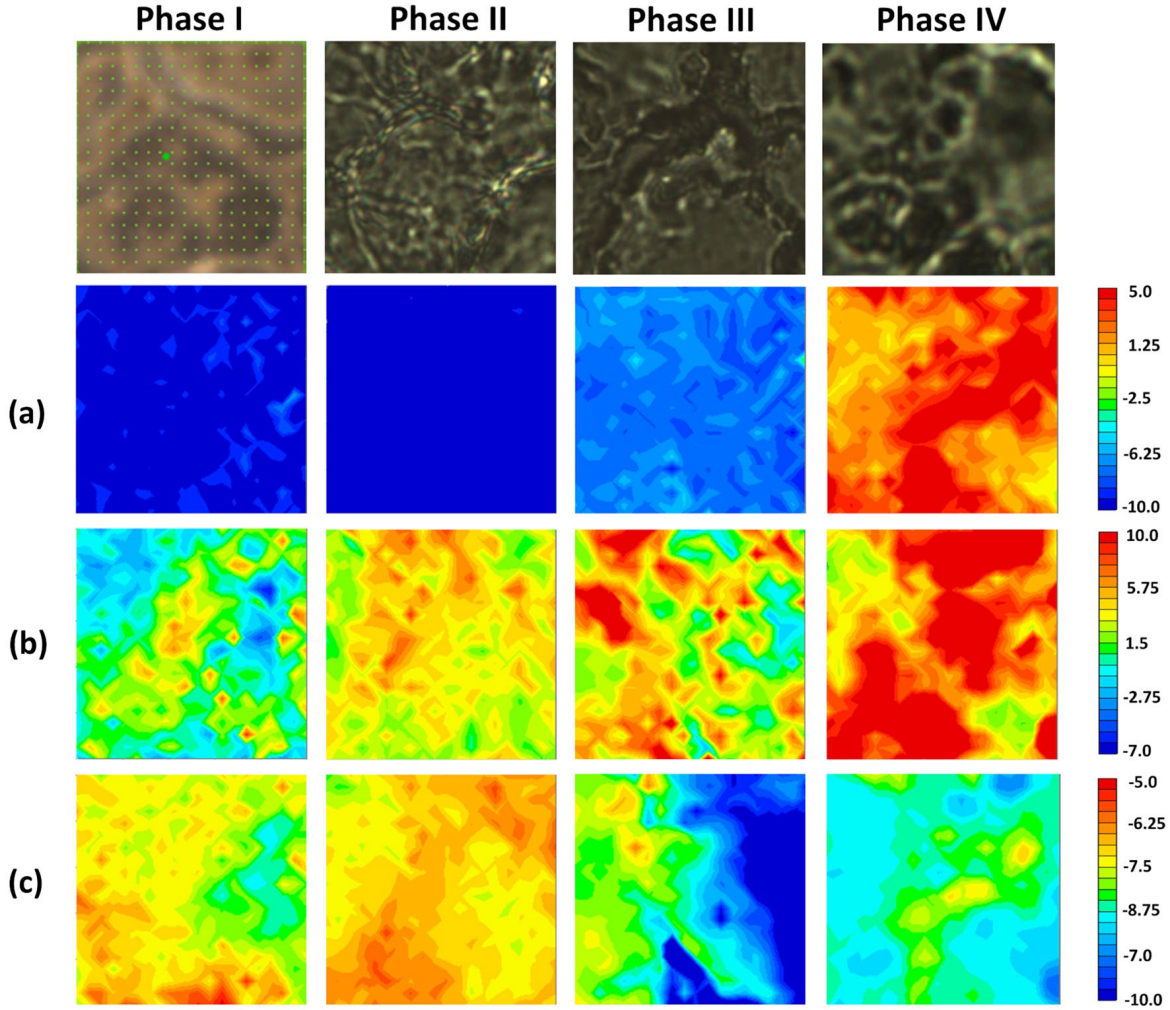
Visible images of cells at each Phase and Raman imaging with a 20 μm $\times$ 20 μm area for fixed cells for the four phases obtained by plotting (**a**) the band intensity at 673 cm^−1^ for the C-S-C stretching vibration, PLS scores for the factor corresponding to the characteristic component of (**b**) fatty acids with a low grade of unsaturation and (**c**) glycoproteins. The visible images with wide area are shown in Fig. S6.

The images in Fig. 4a are plots of the band intensity at 673 cm^−1^ for the C-S-C stretching vibrational mode. The C-S-C component was faintly detected in the image of the cells during Phase III and was widely and densely distributed for Phase IV. The C-S-C component that increased in concentration with cell differentiation could be used to detect the DMSO taken up into cells from the culture medium. DMSO changes the epigenetics of cells and promotes the differentiation of cells from iPSCs to EPO-producing cells. Thus, the variation in the C-S-C band intensity in Fig. 4a reflects epigenetic changes.

Figure 4b was prepared using the scores of Factor 1 (Fig. 2) obtained using the PLS regression analysis performed on artificial increases in the molecular concentration with cell differentiation. The loading plot of Factor 1 exhibited the characteristic spectral pattern for fatty acids with a low degree of unsaturation, i.e., oleic acid. Thus, Fig. 4b shows the variation in the distribution of the unsaturated fatty acids, which clearly increased in concentration with cell differentiation. Namely, the metabolism of fatty acids was found to change. These results demonstrate the considerable potential for using the dynamics of fatty acid metabolism to monitor cell differentiation and indicate candidates for fatty acid species that change in concentration with cell differentiation, such as oleic acid.

Figure 4c was built by plotting the scores of PLSR in two dimensions, where Factor 1ʹ was identified as exhibiting the highest concentration during Phase II and it exhibited the same spectral pattern as that of Factor 2 in Fig. 2. As the loading plot of Factor 1ʹ exhibited the spectral pattern of glycoproteins, Fig. 4c clearly showed the distribution of glycoproteins. The concentration of glycoproteins during Phase II was confirmed to be higher than those during Phases III and IV. This result indicates that the concentration of the glycoproteins that were raw materials for EPO decreased at Phase III and Phase IV because of the release of EPO. Namely, the decrease in the concentration of intracellular glycoproteins could be used as a discriminating factor for the degree of maturation of EPO-producing cells.

## Conclusion

Cell differentiation from iPSCs to EPO-producing cells was investigated using Raman spectroscopy and imaging. The detection of dramatic changes in intracellular molecular compositions over the course of cell differentiation constitutes an enormous advance. The uptake of DMSO by differentiating cells after Phase III was detected and the metabolism of unsaturated fatty acids of EPO-producing cells was found to differ from that of iPS cells. The decrease in the concentration of glycoproteins during Phase III and Phase IV was also made clear.

The results of the present study are a first step towards establishing a method for monitoring EPO produced during the differentiation of cultured iPSC cells as a novel physiological therapy for renal anemia. Feedback on cell dynamics can be used to further the acquisition of cytological findings and update cell culture technology.

## Supplementary Information

Supplementary Information.

## Data Availability

All data used in this study are included in the published article and Supporting Information.
