# Supplementary material for: Exposing intracellular molecular changes during the differentiation of human-induced pluripotent stem cells into erythropoietin-producing cells using Raman spectroscopy and imaging
Source: Sci Rep. 2022 Nov 28;12:20454. doi: 10.1038/s41598-022-24725-1 (PMC9705388; doi:10.1038/s41598-022-24725-1)
Supplement: Supplementary file 1 — Supplementary Information. [file 41598_2022_24725_MOESM1_ESM.pdf]

Supporting Information

Exposing intracellular molecular changes during the differentiation of human-induced pluripotent stem cells into erythropoietin-producing cells using Raman spectroscopy and imaging

Mika Ishigaki[1,2]*, Hirofumi Hitomi[3], Yukihiro Ozaki[4], Akira Nishiyama[5]

[1]*Institute of Agricultural and Life Sciences, Academic Assembly, Shimane University, 1060 Nishikawatsu, Matsue, Shimane, 690-8504, Japan*
[2]*Raman Project Center for Medical and Biological Applications, Shimane University, 1060 Nishikawatsu, Matsue, Shimane 690-8504, Japan*
[3]*Department of iPS Stem Cell Regenerative Medicine, Kansai Medical University, 2-5-1 Shin-machi, Hirakata City, Osaka, 573-1010, Japan*
[4]*School of Biological and Environmental Sciences, Kwansei Gakuin University, 1 Gakuen-Uegahara, Sanda, Hyogo 669-1330, Japan*
[5]*Department of Pharmacology, Faculty of Medicine, Kagawa University, 1750-1 Miki-cho, Kita, Kagawa, 761-0793, Japan*

*Authors to whom correspondence should be sent.
*E-mail: ishigaki@life.shimane-u.ac.jp (M.I.)

Table of Contents

SI 1. Information about cell culture
SI 2. Supporting Raman spectra

SI 1. Information about cell culture

Table S1 shows the substances required for differentiation of hiPSCs into EPO-producing cells, which varied with the cell stages ranging from iPSC cells to Stage 2 (Fig. 1Sa). The cells were incubated under 5% CO_2 at 37°C. In the present study, the cells were fixed for four phases: Phases I, II, III, and IV (Fig. 1Sa). The cells were iPSCs during Phase I and had differentiated into EPO-producing cells by the final stage, Phase IV (10 days after beginning of the cell differentiation from iPSCs). In addition to the phases in which the cells were iPSCs (Phase I) and EPO-producing cells (Phase IV), the cells were also fixed at two intermediate points between Phase I and IV, that is, Phase II and III, when they were 3 and 5 days after beginning of the cell differentiation from iPSCs, and they were differentiated into endoderm cells and hepatic progenitors, respectively. During Phase II, the cells had almost differentiated into endoderm cells. Images of the cells during the four phases are shown in Fig. 1Sb-1Sd.

Cell fixation was carried out by adding a 4% paraformaldehyde solution to the culture medium, which was allowed to stand for approximately 1 h, followed by washing twice with PBS, and drying. An immunostained image of the cells during Phase IV obtained using anti-hEPO (primary antibody), Alexa 594 (secondary antibody), and Hoechst proved that EPO was secreted because the cells were dyed red (Figure 1Se). The detailed procedure for the public protocol was provided by the Center for iPS Cell Research and Application, Kyoto University[20], and the revalidation of the method was reported in our previous research[9].

SI 2. Supporting Raman spectra

The normalization method for Raman spectra based on the band intensity at 1001 cm^{-1} of phenylalanine has been widely used[S1-S3]. In addition, two methods for normalization can be listed as examples of the other major methods, in which the integral of the band at approximately 1450 cm^{-1} due to C-H deformation[S4-S6] or those over the fingerprint region[S7-S9]. In these methods, Raman data are analyzed under the assumption that the concentrations of the representative substances defined as the standards remain constant. Since the uptake of DMSO by cells was detected with their differentiation in this study, the assumptions clearly do not come into effect for the latter two methods. Thus, the normalization by the band intensity due

to phenylalanine was chosen as the best for this study.

The detailed assignment of the Raman bands obtained from fixed cells in Fig. 1 is summarized in Table S2. Figure S2 depicts Raman spectra in the 1800-600 cm^{-1} region for DMSO (043-07216, FUJIFILM Wako Pure Chemical Co., Japan). The band assignment of DMSO spectrum is referred of the reported manuscript[24, 25]. The bands at 1414 and 948 cm^{-1} are assigned to CH_3 bending and CH_3 stretching vibrational modes, respectively. and the one at 1016 cm^{-1} is associated with S=O stretching vibration. The doublet peaks at 702 and 672 cm^{-1} are due to the antisymmetric and symmetric C-S-C stretching vibrations, respectively.

Raman spectra of cysteine and methionine aqueous solutions are shown in Fig. S3. The Raman spectra of water was subtracted as a background. The amino acids aqueous solutions were prepared by dissolving cysteine (039-20652, FUJIFILM Wako Pure Chemical Co., Japan) and methionine (133-01602, FUJIFILM Wako Pure Chemical Co., Japan) in ultrapure water to achieve 200 mM concentrations. The peaks in the 750-600 cm^{-1} are due to C-S stretching vibrational modes[22,33].

Figure S4 shows Raman spectra of 18:n (n=0-3) series of fatty acid: stearic acid, oleic acid, linoleic acid, and linolenic acid. Stearic acid was measured while heating at 80 ℃ to measure it in a liquid state. All spectra were normalized by the peak intensity at approximately 1450 cm^{-1}. Band assignment of fatty acids is summarized in Table S3.

Figure S5 shows the 1800–600 cm^{-1} region of the Raman spectra of EPO powder and EPO aqueous solution. Band assignments for Raman spectra obtained from glucose and glycogen aqueous solutions is shown in Table S4.

References

S1) Avni, A., Joshi, A., Walimbe, A., Pattanashetty, S. G., & Mukhopadhyay, S. (2022). Single-droplet surface-enhanced Raman scattering decodes the molecular determinants of liquid-liquid phase separation. Nature communications, 13(1), 1-13.

S2) Ichimura, T., Chiu, L. D., Fujita, K., Machiyama, H., Kawata, S., Watanabe, T. M., & Fujita, H. (2015). Visualizing the appearance and disappearance of the attractor of differentiation using Raman spectral imaging. Scientific reports, 5(1), 1-10.

S3) Howell, N. K., Herman, H., & Li-Chan, E. C. (2001). Elucidation of protein− lipid interactions in a lysozyme− corn oil system by Fourier transform Raman spectroscopy. Journal of Agricultural and Food Chemistry, 49(3), 1529-1533.

S4) Short, K. W., Carpenter, S., Freyer, J. P., & Mourant, J. R. (2005). Raman spectroscopy detects biochemical changes due to proliferation in mammalian cell cultures. *Biophysical journal*, *88*(6), 4274-4288.

S5) Wang, P. J., Ferralis, N., Conway, C., Grossman, J. C., & Edelman, E. R. (2018). Strain-induced accelerated asymmetric spatial degradation of polymeric vascular scaffolds. *Proceedings of the National Academy of Sciences*, *115*(11), 2640-2645.

S6) Motz, J. T., Hunter, M., Galindo, L. H., Gardecki, J. A., Kramer, J. R., Dasari, R. R., & Feld, M. S. (2004). Optical fiber probe for biomedical Raman spectroscopy. *Applied optics*, *43*(3), 542-554.

S7) Huang, Z., McWilliams, A., Lui, H., McLean, D. I., Lam, S., & Zeng, H. (2003). Near-infrared Raman spectroscopy for optical diagnosis of lung cancer. *International journal of cancer*, *107*(6), 1047-1052.

S8) Zhao, J., Zeng, H., Kalia, S., & Lui, H. (2016). Wavenumber selection based analysis in Raman spectroscopy improves skin cancer diagnostic specificity. *Analyst*, *141*(3), 1034-1043.

S9) Lau, D. P., Huang, Z., Lui, H., Man, C. S., Berean, K., Morrison, M. D., & Zeng, H. (2003). Raman spectroscopy for optical diagnosis in normal and cancerous tissue of the nasopharynx—preliminary findings. *Lasers in surgery and medicine*, *32*(3), 210-214.

Table S1: The substances used to induce differentiation of hiPSCs into EPO-producing cells during different cell stages.

	**Stage 0 (iPS)**	**Stage 1**	**Stage 2**
**Factor**	10 μM Y-27632	1 μM CHIR99021<br>Activin A 100 ng/ml	1% DMSO
**50 ml of Medium**	iPS on Matrigel	49 ml RPMI 1640<br>1 ml B27	38.5 ml KO-DMEM<br>10 ml KSR<br>0.5 ml L-glutamine<br>0.5 ml NEAA<br>0.1 ml β-mercaptoethanol

Table S2: Band assignments for Raman spectra obtained for fixed iPSCs.

Band (cm^{-1})	DNA/RNA	proteins	lipids	carbohydrates
1737			C=O str	
1656-1657		Amide I	C=C str	
1443-1447		CH def	CH def	CH def
1337-1300		CH_3/CH_2 twi, ben	CH_3/CH_2 twi, ben	
1260-1240	T, A	Amide III	=C-H ben	
1124		C-N str		C-O str
1060	PO_2^- str			C-C str, C-O str
1030		C-H ben Phe		C-C str
1001		sym ring br Phe		
948				C-C str
850		ring br Tyr		
824	PO_2^- asym str	ring br Tyr		
780	U, T, C			
718			$CN^+(CH_3)_3$	

Abbreviation: br: breathing, str: stretching, twi: twisting, ben: bending, def: deformation, sym: symmetric, asym: asymmetric

Table S3: Band assignment of fatty acids[22, 35-37].

Band position	Assignment
1750-1730	C=O str
1680-1640	C=C str
1500-1400	C-H def
1305-1295	CH_3/CH_2 twi, ben
1280-1250	=C-H ben

Table S4: Band assignments for Raman spectra obtained from glucose and glycogen aqueous solutions[38-40].

D-glucose		glycogen	
Band (cm^{-1})	Assignment	Band (cm^{-1})	Assignment
1460	$\delta(CH_2)$	1458	$\delta(CH_2)$, β(COH)
1368	$\omega(CH_2)$	1378	$\beta(CH_2)$
1333	$\omega(CH_2)$	1336	β(COH), β(CH)
1267	$\tau(CH_2)$	1260	β(CH), β(CCH), β(OCH), β(COH)
1122	ν(CC), ν(CO), β(COH)	1124	ν(CC), ν(CO), β(COH), ν_s(COC) in glycosidic link, ring br
1061	ν(CC), ν(CO), β(COH)	1080	β(COH)
912	β(CCH), β(CCO)	937	ν_s(COC) of α-D-(1-6) glycosidic linkages, ring def
860	ν(CC)	860	ν(COC), ring br

ν: stretching, s: symmetric, β: in-plane bending, δ: scissoring, ω: wagging, τ: twisting, def: deformation, br: breathing

(a)

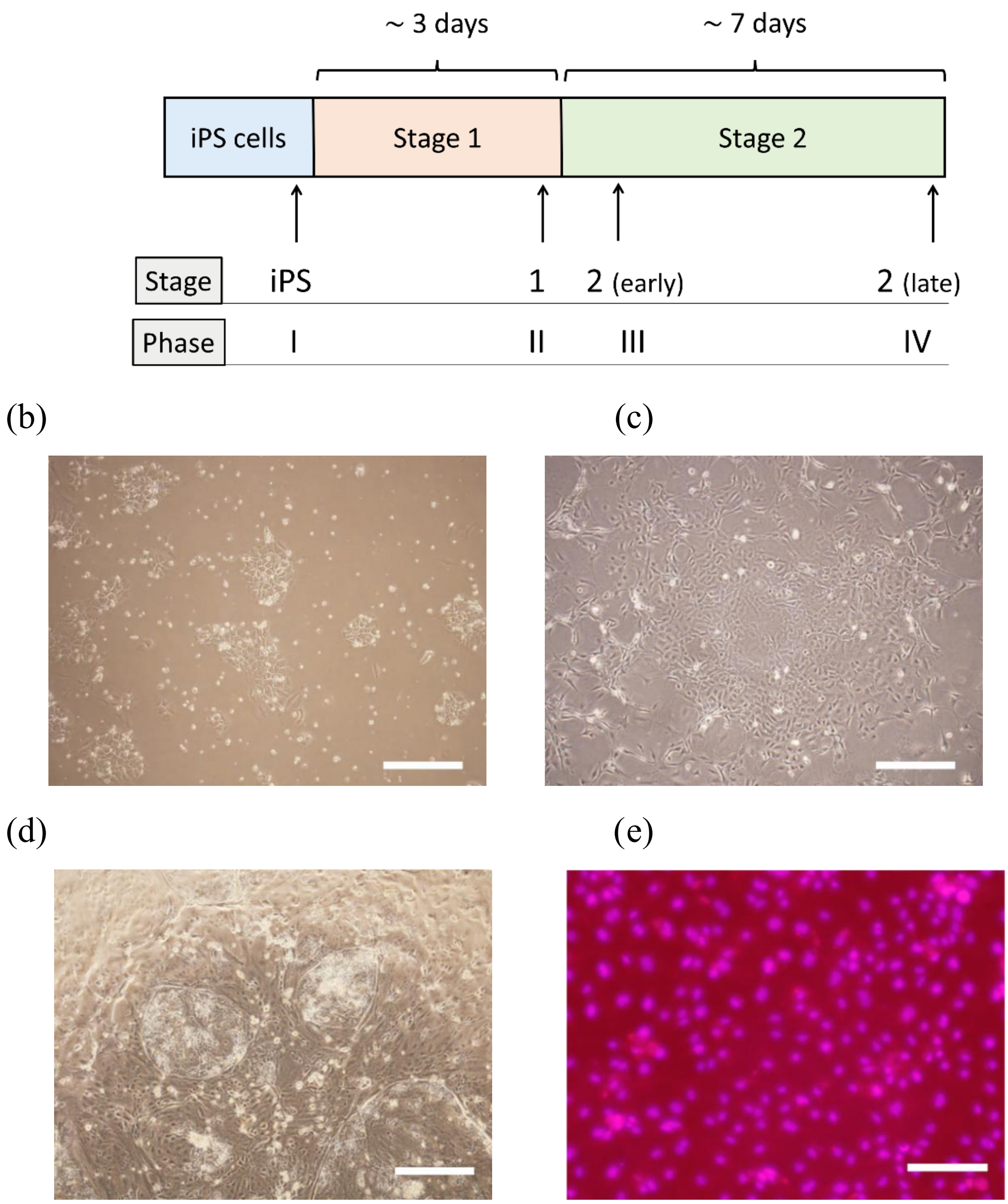

Figure S1: (a) The experimental design for cell fixation during the course of cell differentiation from hiPSCs into EPO-producing cells. Visible images of the cells at Phases (b) I, (c) III, and (d) IV. (e) Image obtained by immunostaining with anti-hEPO, Alexa 594, and Hoechst for Phase IV. Scale bars express 50 μm.

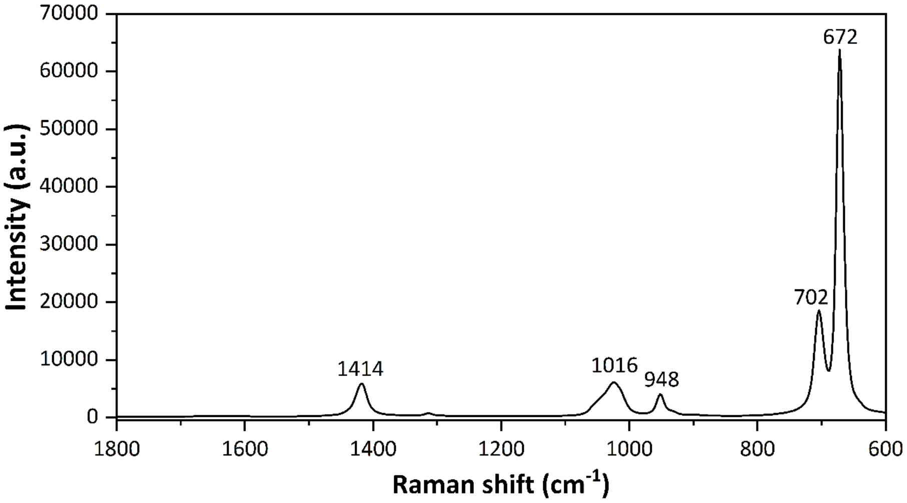

Figure S2: The 1800–600 cm^{-1} region of the Raman spectra for DMSO obtained under a 532-nm excitation.

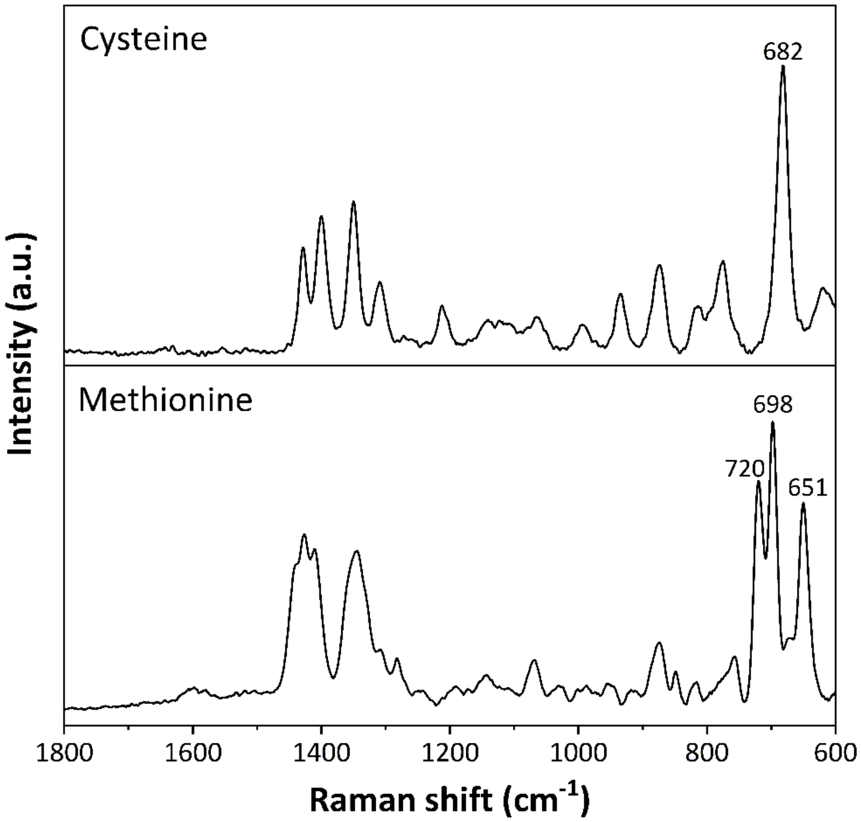

Figure S3: The 1800–800 cm^{-1} region of the Raman spectra for 200 mM cysteine and methionine aqueous solutions.

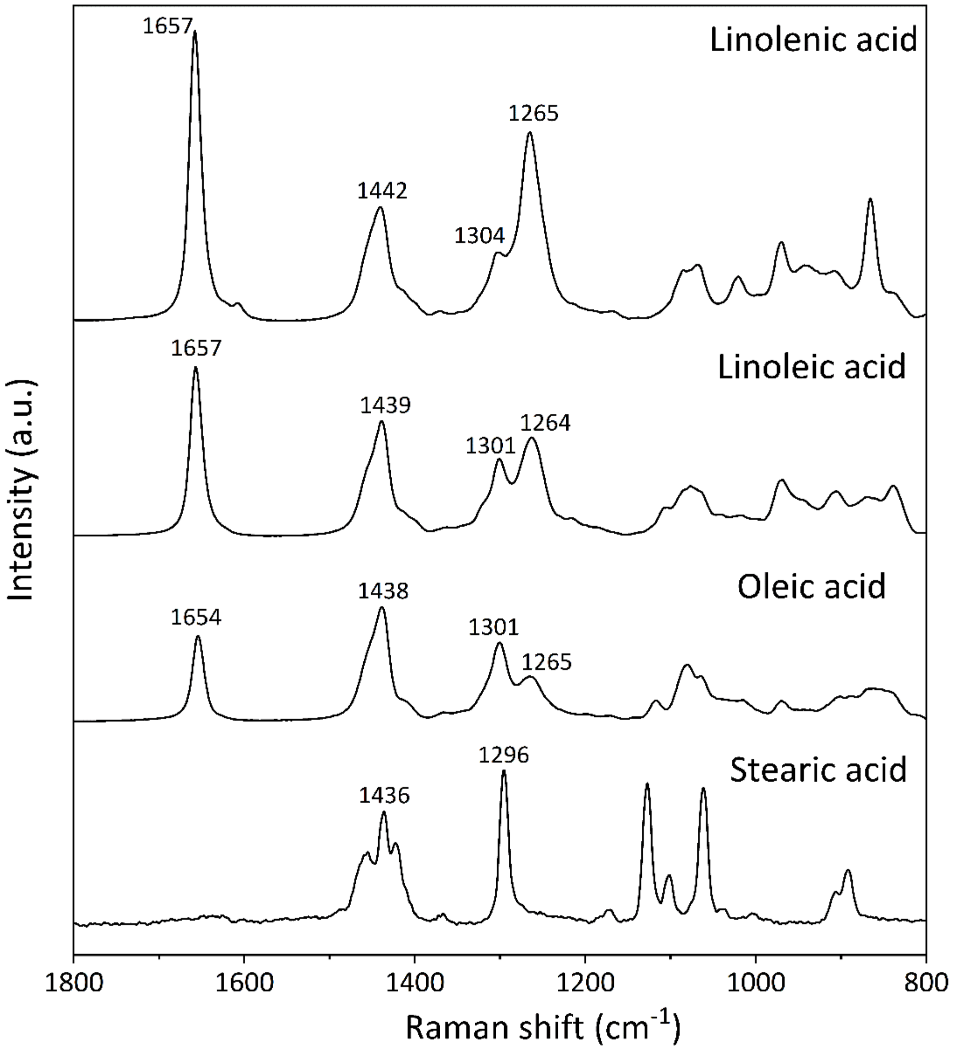

Figure S4: The 1800–800 cm^{-1} region of the Raman spectra in for an 18:n series of fatty acids in a liquid state.

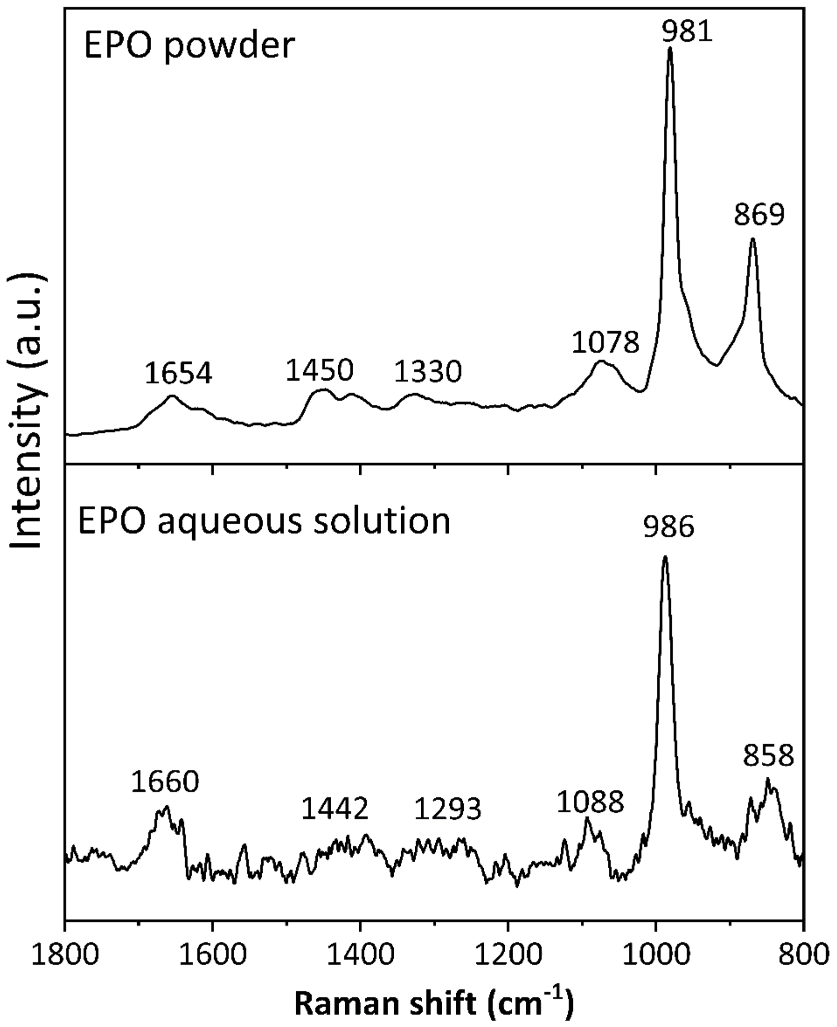

Figure S5: The 1800–600 cm^{-1} region of the Raman spectra for EPO powder and EPO aqueous solution.

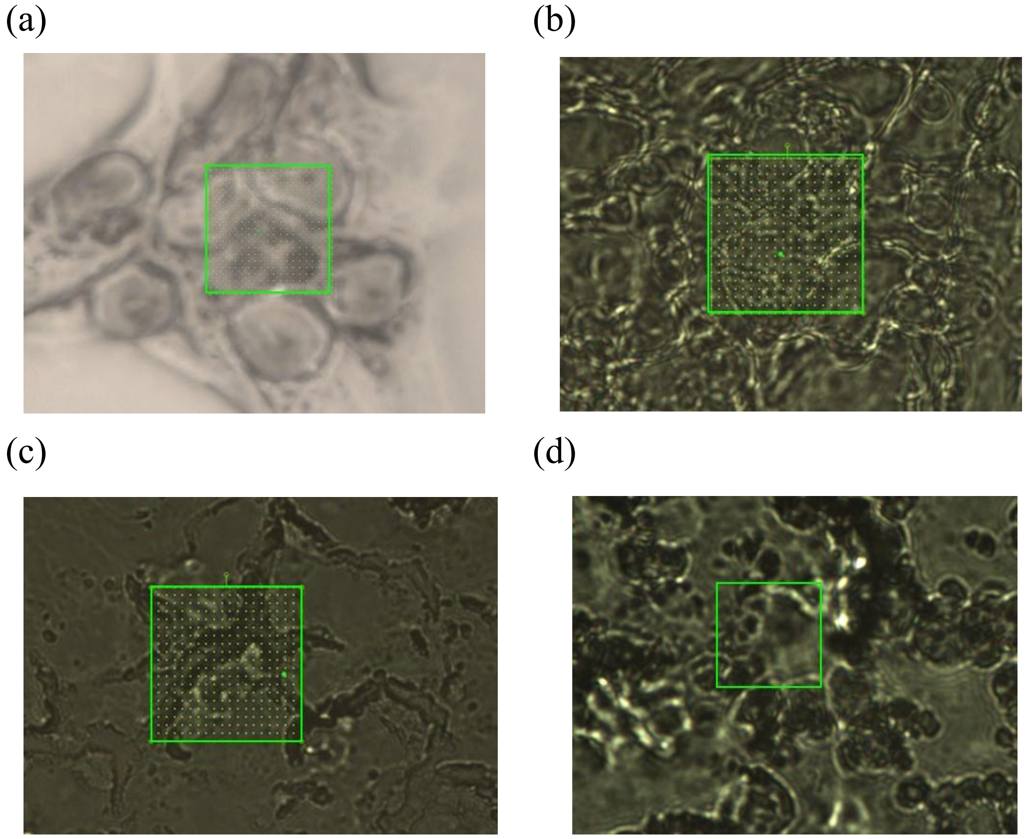

Figure S6: The visible images of Figure 4 for (a) Phase I, (b) Phase II, (c) Phase III, and (d) Phase IV with wide area. The yellow-green squares exhibit the measurement areas for Raman imaging with 20 μm × 20 μm.
